# 739. The High Burden of Bloodstream Infection in Adults: A Nation-Wide Population- Based Study

**DOI:** 10.1093/ofid/ofac492.030

**Published:** 2022-12-15

**Authors:** Vered Schechner, Liat Wullfhart, Elizabeth Temkin, Sarah F Feldman, Amir Nutman, Pnina Shitrit, Mitchell J Schwaber, Yehuda Carmeli

**Affiliations:** Israel Ministry of Health, Tel Aviv, Tel Aviv, Israel; Israel Ministry of Health, Tel Aviv, Tel Aviv, Israel; Israel Ministry of Health, Tel Aviv, Tel Aviv, Israel; Israel Ministry of Health, Tel Aviv, Tel Aviv, Israel; Israel Ministry of Health, Tel Aviv, Tel Aviv, Israel; Israel Ministry of Health, Tel Aviv, Tel Aviv, Israel; Israel Ministry of Health, Tel Aviv, Tel Aviv, Israel; Israel Ministry of Health, Tel Aviv, Tel Aviv, Israel

## Abstract

**Background:**

Limited data exist on the long-term consequences of bloodstream infections (BSI). We examined incidence, 1-year mortality, and years of potential life lost (YPLL) following BSI by eight sentinel bacteria in adults. We also estimated the relative contribution of hospital-onset BSI (HO-BSI) and of antibiotic-resistant BSI to the burden of BSI.

**Methods:**

We used data from Israel's national BSI surveillance system (covering *E. coli*, *K. pneumoniae*, *P. aeruginosa*, *A. baumannii*, *S. pneumoniae*, *S. aureus*, *E. faecalis* and *E. faecium*, comprising 70% of all BSI) and the national death registry. Adults with BSI between 1/1/2018 and 31/12/2019 were included. The outcomes were all-cause 30-day and 1-year mortality. We constructed Kaplan-Meier curves and used the log-rank test to compare survival between age groups. We calculated the age-standardized mortality rate and YPLL using the Global Burden of Disease reference population and life expectancy tables.

**Results:**

A total of 25,376 BSI occurred over 2 years in the adult Israeli population (mean adult population: 6,068,580). Drug-resistant BSI comprised 32% of all events (41.1% of HO-BSI). The annual BSI incidence was 209 per 100,000 population. The case fatality rate (CFR) was 25.6% at 30 days and 46.4% at 1 year. The 1-year CFR was higher among males than females (RR=1.17, 95% CI 1.14–1.20) and it increased with age (OR=1.37 per decade, 95% CI 1.36–1.39). In all age groups survival continued to decline up to 1 year (Figure). For the outcome of 1-year mortality, the annual age-standardized mortality rate and YPLL per 100,000 population following BSI were 48.6 and 1002.2, respectively. For comparison, the global mortality rate and YPLL from ischemic stroke are 36.6 and 521.8 per 100,000, respectively. HO-BSI represented 27.4% of BSI but contributed to 33.9% of deaths and 39.9% of YPLL. HO-BSI by drug-resistant bacteria represented 11.3% of BSI, 14.8% of deaths, and 17.4% of YPLL.

**Conclusion:**

BSI have grave long-term consequences: nearly 50% of affected adult patients died within 1 year. The burden of BSI is higher than that of ischemic stroke. HO-BSI and drug-resistant BSI contribute disproportionately to BSI mortality and YPLL. Attention and resources should be directed to prevention of BSI, as well as to advances in early diagnosis and better treatment.

**Disclosures:**

**Yehuda Carmeli, MD**, Allecra Therapeutics: Advisor/Consultant|MSD: Advisor/Consultant|Nabriva: Advisor/Consultant|Pfizer: Advisor/Consultant|Qpex Pharmaceuticals: Advisor/Consultant|Roche: Advisor/Consultant|Shinogi: Advisor/Consultant|Spero Therapeutics: Advisor/Consultant.

**Figure.**
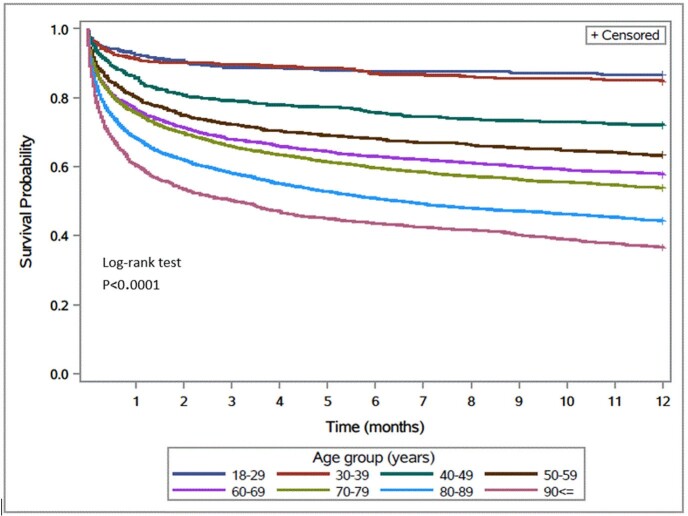
One-year survival following BSI by eight sentinel bacteria, by age, Israel, 2018–2019

